# Supplementary material for: Super-Resolution Analysis of the Origins of the Elementary Events of ER Calcium Release in Dorsal Root Ganglion Neurons
Source: Cells. 2023 Dec 23;13(1):38. doi: 10.3390/cells13010038 (PMC10778190; doi:10.3390/cells13010038)
Supplement: Supplementary file 1 [file cells-13-00038-s001.zip › cells-2755251-supplementary.pdf]

cells MDPI

Supporting Information

1. Supplementary methods

1.1. 10x Expansion Microscopy sample preparation

Immunolabelled samples were incubated with 0.1 mg/ml acryloyl-X (Thermo Fisher Scientific) in PBS overnight at 4°C, then washed in PBS immediately prior to addition of gel solution.

X10 ExM was performed on DRG and glial cell co-cultures that had been fixed and immunofluorescence stained, similar to the dSTORM staining protocol. For ExM, goat anti-rabbit Alexa Fluor 488 secondary antibodies were chosen. X10 gels (4:1 molar ratio of dimethylacrylamide (Sigma-Aldrich) and sodium acrylate (Sigma-Aldrich), dissolved in deionized H_20 (dH_2O)) were made according to the previous recipe. Gel solution was made fresh and bubbled with nitrogen gas for one hour on ice. Potassium persulfate (Sigma-Aldrich) was added from a fresh 0.036 g/ml stock to 0.4% molar relative to the monomer concentration and the solution was bubbled for another 15 mins on ice. 500 µl of the gel solution was mixed rapidly with 2 µl of N,N,N',N'-Tetramethylethylenediamine (Sigma-Aldrich) and quickly added to the sample coverslip. The polymerisation chamber, comprising the sample coverslip with two coverslip spacers either side, was sealed with a top coverslip. Gels were polymerised after two hours. The major axes of the gel were measured to calculate the pre-expansion size.

Polymerised gels were removed from the coverslip chamber and placed into 6-well plates to undergo digestion in 0.2 mg/ml proteinase K (New England Biolabs) dissolved in digestion buffer (50 mM Tris pH 8.0 (Thermo Fisher Scientific), 1mM ethylenediaminetetraacetic acid (Sigma-Aldrich), 0.5% Triton X-100, 0.8M guanidine HCl (Sigma-Aldrich) and dH_20) overnight at room temperature. Gels were expanded by shaking in dH_20 until the gel expansion reached a plateau, replacing the dH_20 every hour. The final gel size was measured to calculate the macroscale expansion factor, in relation to the pre-expansion size.

1.2. 'Enhanced ExM' image acquisition

Expanded gels were placed into acrylic chambers with a square cut-out, attached to a no. 1.5 glass coverslip (Menzel Gläser), which had been coated with 0.1% (v/v) poly-L-lysine (Sigma-Aldrich) at room temperature for 30 minutes. Airyscan imaging was performed on an inverted LSM880 (Carl Zeiss, Jena), with either a Plan-Aprochromat 40x 1.3 NA objective or a Plan-Apochromat 20x 0.8 NA objective lens. AlexaFluor 488 fluorophores were excited with 488 nm DPSS laser, while emission band was selected using the in-built spectral detector. Airyscan datasets were subjected to a pixel reassignment and a linear deconvolution *via* ZEN suite (Zeiss).

1.3. Simulations

1.3.1. Simulation of confocal images from dSTORM data

To interrogate which features of the punctate labelling densities observed in RyR and IP3R1 super-resolution image data would not be resolved under diffraction-limited imaging, we simulated the equivalent confocal images from the 10x EExM data. Image data that were scale adjusted according to the estimated Expansion Factor were convolved with a two-dimensional Gaussian function with a FWHM using ImageJ. Supplementary Figure S2 illustrates the before and after comparison.

1.3.2. Resolution and Signal-to-noise ratio on alignment

For simulating the impact of resolution and the signal-to-noise ratio (SNR), we simulated an averaged Ca^{2+} spark image based on an experimentally recorded and rendered

dSTORM image of RyR on a DRG. The simulated Ca^{2+} spark image was randomly misaligned and then realigned using the alignment code. To investigate the impact of SNR on the alignment, the Ca^{2+} image, traced from the dSTORM image, was convolved by a 2D Gaussian function with a FWHM of 250 nm and then noise added such that the SNR would vary between 5.19 and 29.26 using the freely available ImageJ plugin, SNR, developed by Dr Daniel Sage [43] (http://bigwww.epfl.ch/sage/soft/snr/). The same plugin was used for estimating the SNR of the typical experimental, averaged Ca^{2+} spark image (typically 15.5-18.0), as a reference point. In each of 10 iterations of re-alignment, the difference in each of the x and y coordinates was recorded as the alignment error and used for plotting Fig S4B. For simulating the impact of resolution, the simulated Ca^{2+} image, traced from the dSTORM image, was convolved with 2D Gaussian functions varying between 50 and 1000 nm. Noise was added to each simulated image to achieve an SNR of 17.0 before subjecting it to the same re-alignment iterations. Fig S4A show the violin plots of the alignment error in each of x and y dimensions after 10 iterations.

1.4. Super-resolution image-based statistics and resolution test

1.4.1. Resolution test for dSTORM data

dSTORM localisation datasets for RyR were used to estimate the true resolution achieved in these images. Datasets (in .h5r format) were opened using the PYME software and sub-sampled into two groups – one consisting of events in even-numbered frames and one consisting odd numbered frames. The two images were rendered onto greyscale, 16-bit TIFF images at a pixel sampling of 5 nm/pixel using the *Render using Jittered Triangulation* function (with the default settings). The two images were imported to ImageJ and pixel sampling was inputted using the *Set Scale* function. Residual offset values were subtracted from each image using the *Subtract* function. A Fourier Ring Correlation analysis (shown in Supplementary Figure S1) was then performed with the *FRC* plugin made available with the BioImaging And Optics Platform (BIOP) plugin suite; https://github.com/BIOP/ijp-frc. The 1/7 of the peak correlation fixed threshold was selected as the criterion for determining the minimum correlated for Fourier Image Resolution (FIRE) as defined by Nieuwenhuizen et al. [44]

1.4.2. Puncta size analysis

To examine the impact of resolution on the visualisation of the RyR and IP3R1 puncta, the dSTORM and 10x EExM image data were subjected to particle size analysis. Briefly, the images were rendered onto 16-bit TIFF image formats and imported into ImageJ and the correct pixel sampling was inputted. For 10xEExM, the pixel sampling value was divided by the Expansion Factor estimated by measuring the dimensions of the gel before and after expansion. A binary mask of each image was created by subjecting it to *Otsu* thresholding (found under the *Auto Thresholding* routines). The *Analyse Particles* function was then used to export the areas of each punctum area in the binary mask into a list that was copied over to Prism v9. Puncta area measurements from multiple image data sets were pooled to create the percentage histograms of the puncta area (see details in Supplementary Figure S2).

2. Supplementary Figures

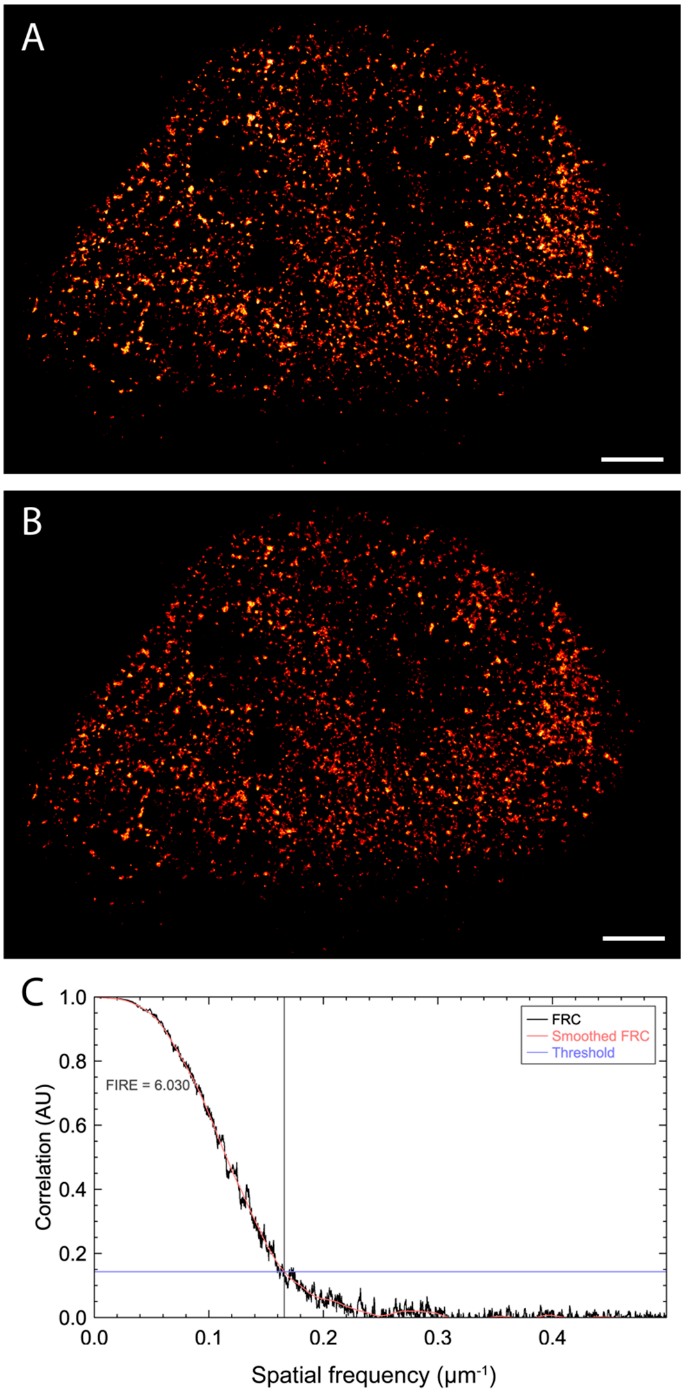

Figure S1. Fourier ring correlation analysis to estimate resolution in dSTORM localisation data of RyR. For this analysis, the localisation point data were randomly split into two sub-samples and rendered into two greyscale images (A&B) with a pixel sampling of 5 nm/pixel using the software PYME. Scale bars: 2 μm. Fourier Ring Correlation Plugin for ImageJ (based on the analysis proposed by Nieuwenhuizen et al. [44]). C. Shown, is the Fourier Ring Correlation (black solid line), smoothed version of the same plot (red line) and the 1/7 of the peak correlation fixed threshold line (blue), plotted as a function of the spatial frequency. The smoothed correlation line intersects with the threshold line at spatial frequency of ~ 1.677 μm^{-1} which translates to the smallest correlated length scale of ~ 30 nm.

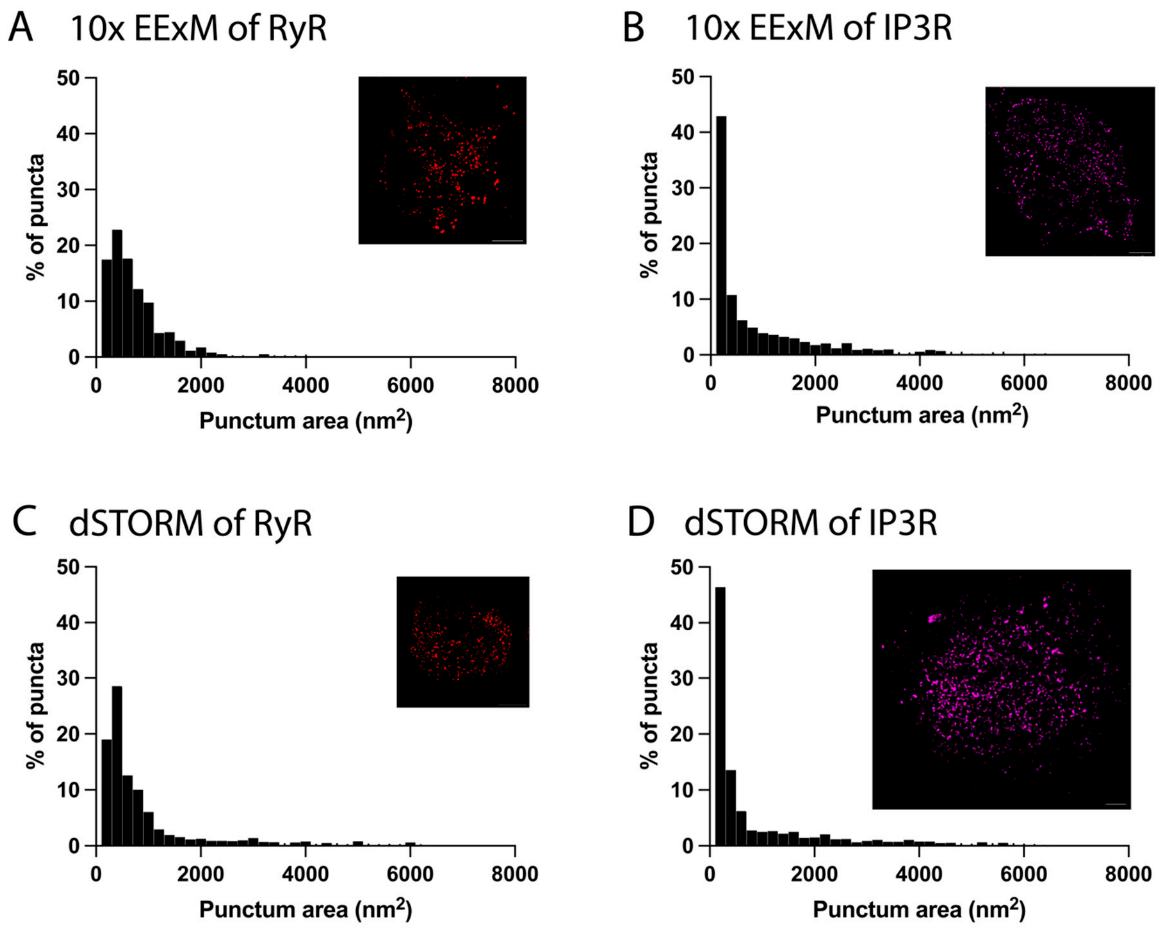

Figure S2. Puncta area analysis of RyR and IP3R1 images acquired with dSTORM and 10x EExM. Shown, are percentage histograms of (A) RyR puncta (n=1666 puncta from 4 cells) and (B) IP3R1 puncta area (n=1452 puncta from 3 cells) in 10x EExM. These are compared with (C) RyR puncta (n=5224 puncta from 6 cells) and (D) IP3R1 puncta area (n= 1634 puncta from 3 cells) in dSTORM. Insets indicate example images from each analysis; scale bars: 1 µm).

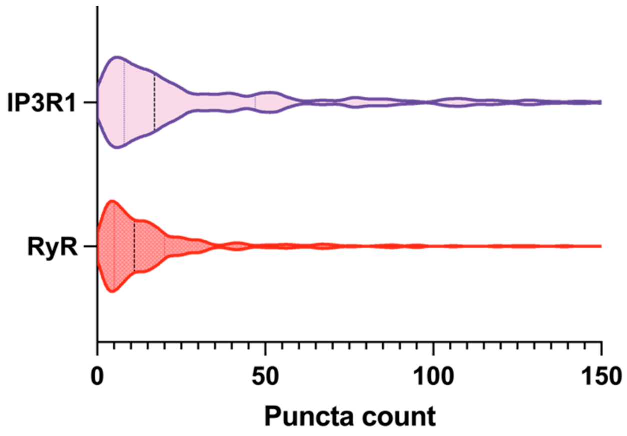

Figure S3. Violin plots of the total puncta counts of IP3R1 (purple) RyR (red) detected underlying each spark footprint. The dashed black line indicates median and dotted lines, upper and lower quartiles. The mean ± SD for IP3R1 and RyR counts were 34.8 ± 41.79 (n = 339 sparks, 7 cells) and 19.4 ± 26.6 puncta (n = 263 sparks, 6 cells) respectively. .

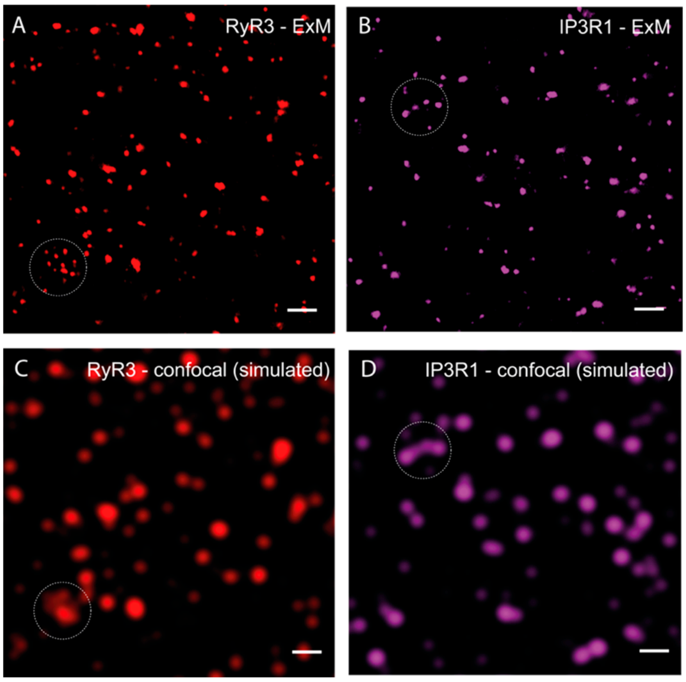

Figure S4. 10x EExM images of RyR and IP3R1 and simulating the confocal images of the resolved morphology. A&B. RyR and IP3R1 immunofluorescence visualised with 10x EExM at an in-plane resolution of ~ 15 nm. C&D. The equivalent confocal image simulated by convolving the ExM image with a confocal point spread function, approximated by a two-dimensional Gaussian with a FWHM of 250 nm. It was clear that at least closely located puncta were either blurred into individual objects or undetected in the simulated confocal images (see equivalent regions indicated by the dashed circles). Scale bars 500 nm.

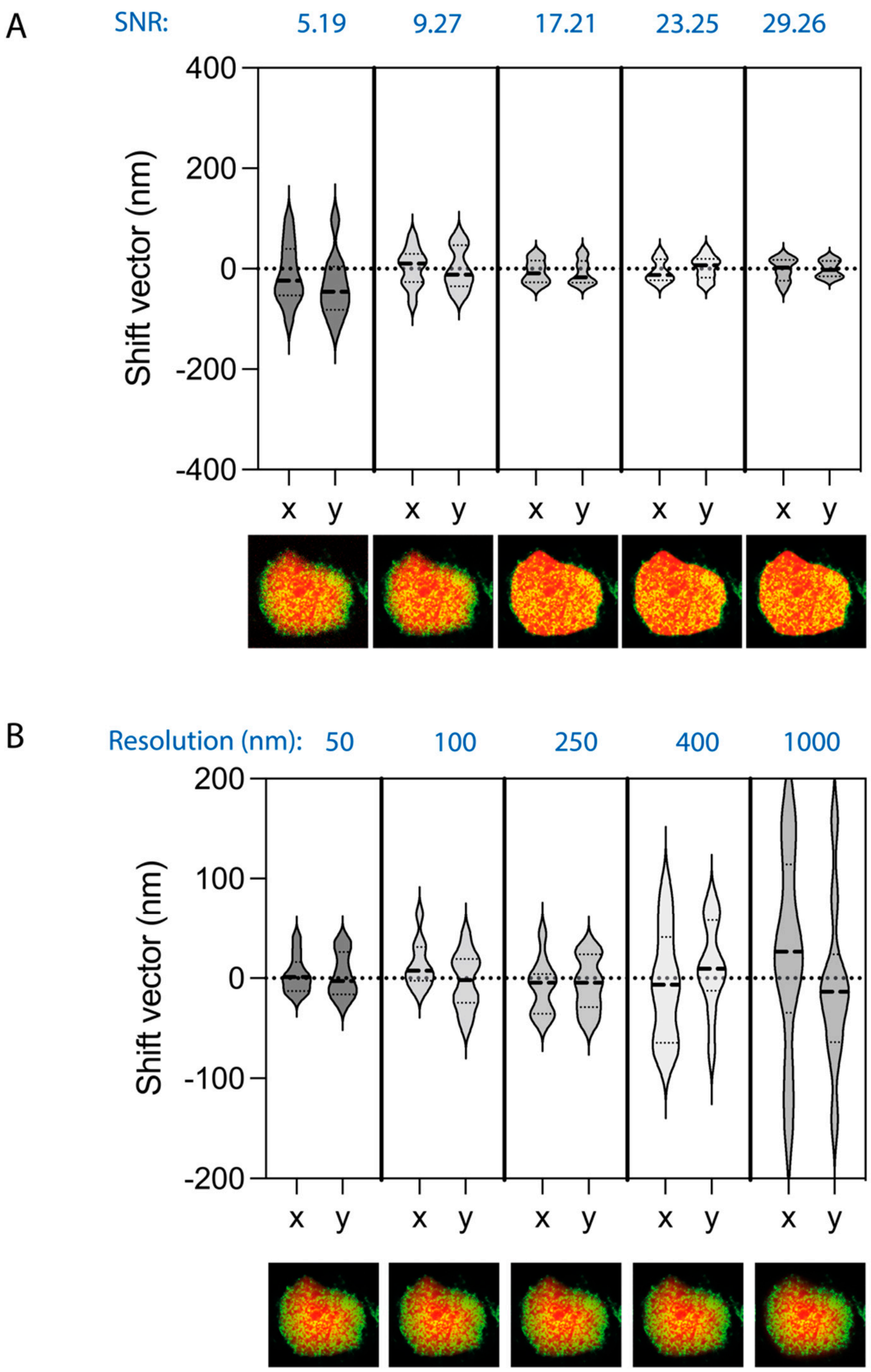

Figure S5. Simulation of the effect of resolution and signal-to-noise-ratio (SNR) on the alignment error. A. Violin plots of the alignment error in the x and y dimensions in iterative alignment of a convolved copy (the FWHM of the convolution point spread function representing the 'Resolution' value indicated) of the simulated Ca^{2+} image against the experimentally mapped RyR dSTORM image. **B.** Violin plots of the alignment error in the x and y dimensions in iterative alignment of a noise-added copy of the simulated diffraction-limited Ca^{2+} image against the experimentally mapped RyR dSTORM image.
